# Supplementary material for: X-ray flares from the stellar tidal disruption by a candidate supermassive black hole binary
Source: Nat Commun. 2020 Nov 18;11:5876. doi: 10.1038/s41467-020-19675-z (PMC7674478; doi:10.1038/s41467-020-19675-z)
Supplement: Supplementary file 1 — Supplementary Information [file 41467_2020_19675_MOESM1_ESM.pdf]

Supplementary Information

X-ray flares from the stellar tidal disruption by a candidate supermassive black hole binary

Xinwen Shu[1*], Wenjie Zhang[1], Shuo Li[2], Ning Jiang[3], Liming Dou[4], Zhen Yan[5], Fu-Guo Xie[5], Rongfeng Shen[6], Luming Sun[1], Fukun Liu[7,8], Tinggui Wang[3]

[1]*Department of Physics, Anhui Normal University, Wuhu, Anhui 241002, China. (*Correspondence to xwshu@ahnu.edu.cn)*
[2]*National Astronomical Observatories, Chinese Academy of Sciences, Beijing 100101, China*
[3]*CAS Key Laboratory for Researches in Galaxies and Cosmology, Department of Astronomy, University of Science and Technology of China, Hefei, Anhui 230026, China*
[4]*Center for Astrophysics, Guangzhou University, Guangzhou 510006, China*
[5]*Shanghai Astronomical Observatory, Chinese Academy of Sciences, Shanghai 200030, China*
[6]*School of Physics and Astronomy, Sun Yat-Sen University, Zhuhai 519082, China*
[7]*Department of Astronomy, Peking University, Beijing 100871, China*
[8]*Kavli Institute for Astronomy and Astrophysics, Peking University, Beijing 100871, China*

Supplementary Note.1—The host galaxy. To derive the physical properties of the host galaxy of OGLE16aaa by modeling the SED, we used the photometric data from GALEX, Swift UVOT, the APM survey, 2MASS, and WISE. We fitted the photometry with stellar population synthesis models using the code `FAST`[1]. To construct the library of stellar population models, we adopted a Chabrier initial mass function, exponentially declining star-formation history with e-folding times in the range $\tau = 0.1 - 10$ Gyr with solar metallicity, standard Calzetti law with a very wide range of dust extinction of $0 < A_V < 6$, and stellar population ages from 0.01 to 10 Gyr. The best-fit template from `FAST` yields a galaxy stellar mass and SFR of log$M_\star$=10.11 $\mathrm{M}_\odot$[9.49–10.22 $\mathrm{M}_\odot$]and 3.7 $\mathrm{M}_\odot\,\mathrm{yr}^{-1}$[2.5–370 $\mathrm{M}_\odot\,\mathrm{yr}^{-1}$], and a total dust extinction of $A_\mathrm{V} = 1.0$ [0.69–2.65], where the values in brackets represent the 68% confidence intervals. The photometric data and best-fit SED template is shown Supplementary Table 2 and Figure 1, respectively. We can estimate the central black hole mass for OGLE16aaa from the stellar mass by using the scaling relation presented in[2], yielding $M_\mathrm{BH} = 3.2^{+1.0}_{-2.5} \times 10^6\,\mathrm{M}_\odot$. This is consistent with that obtained from the UV/optical light curve fittings[3].

Supplementary Note.2—Modeling the photometric light curve for UVW2 and I-band. In addition to the canonical $t^{-5/3}$ decay model ($L = L_0(t - t_0)^{-5/3}$), we have also tried the fittings with an exponential model ($L = L_0 e^{-(t-t_0)/\tau}$) to the UVW2 data. The exponential model describes the data slightly better according to the χ^2 statistics (Supplementary Figure 2, left), perhaps due to the large errors of flux after $t \sim 300$ days. In either case, the tentative evidence of re-brightening in the UVW2 at $t \sim 40$ days is not significant. Removing the data from the period of tentative re-brightening (from $t = 41$ to $t = 54$ days) yields only a slight difference in the reduced χ^2, from χ^2/d.o.f=38.6/21=1.83 to χ^2/d.o.f=30.8/18=1.71, i.e., at a confidence level of about 90% for changing of 3 d.o.f. Similar analysis was performed on the optical I-band data (Supplementary

Figure 2, right). The optical re-brightening appears more evident, as suggested by the data/model plot in the lower panels.

Supplementary Note.3—X-ray luminosity evolution in comparison with other TDEs. The delayed X-ray re-brightening in OGLE16aaa is somewhat similar to that of the TDEs ASASSN-15oi, AT2018fyk, and AT2019azh. For a more detailed comparison, we retrieved and processed all the X-ray data observed with Swift/XRT up to 2020 June, for the three TDEs, with procedures the same as we used for OGLE16aaa. The X-ray luminosity evolution as a function of time is shown in the Supplementary Figure 5. It can bee seen that the X-ray light curve for other TDEs does not show an X-ray brightening as abrupt as in OGLE16aaa. However, due to the lack of data verifying the rise time to peak for other TDEs, such a comparison should be treated with caution.

Supplementary Note.4—Comparison of X-ray spectral properties with the galaxy J1201+3003. SDSS J120136.02+300305.5 (J1201+3003) was noted in the XMM-Newton slew observations due to the detection of a tidal disruption-like X-ray flare[4]. In several aspects J1201+3003 differs from standard TDE candidates discovered in the X-rays. Its unusual X-ray light curve with the strong variations superposed upon the canonical $t^{-5/3}$ evolution is consistent with the prediction of stellar tidal disruption by a supermassive black hole binary[5]. In addition, the X-ray spectra of J1201+3003 are also unusual, which cannot be modeled by a single blackbody emission as originated from a thermal disk, requiring an additional harder component. Such a spectral property appears similar to OGLE16aaa. The results of spectral fittings with the double blackbody emission for both objects are shown in the Supplementary Figure 6.

Supplementary Note.5—Possible origin for the additional spectral component. By investigating in detail the X-ray spectra of the TDEs presented in the Figure 4 of main text, we found that all present an additional X-ray emission component. In order to further investigate on the origin of the additional X-ray component, we performed uniform spectral fittings for these TDEs (and J1201+3003), using a blackbody model plus an extra powerlaw component. The resulting plot of blackbody temperature versus photon index is shown in the Supplementary Figure 7. We found a clear diversity of X-ray spectral properties. Four TDEs have a photon index for the extra powerlaw component consistent with that of typical AGNs[6]. However, we argue that this does not necessarily mean the underlying weak AGN emission as origin for the extra component, because its flux appears to vary with time[7,8]. For AT2018fyk, we also find rapid steeping in the photon index from $\Gamma = 2.3$ to $\Gamma = 4.1$ within only one year, which is atypical in AGNs. For ASASSN14li and OGLE16aaa, the best-fit yields a photon index for the extra component of $\Gamma > 4$, which is rare among AGNs. Only one bona fide AGN of this kind has been reported so far (RX J1302+2747, [9]). Given its rapid flux and spectral variability, one possible origin for the extra component might be a transient corona that is connected with the formation and evolution of accretion disk. The different photon indices observed could be simply explained by the variety in the physical condition of the coronal region. However, since the formation and evolution of hot corona in TDEs are poorly studied so far, such an interpretation for the origin of the additional X-ray component is still tentative, and requires to be tested with future observations of more similar TDEs.

Supplementary Table 1: Swift Observations of OGLE16aaa

obsID	obs. date	days	mag_{UVW2}	mag_{UVM2}	mag_{UVW1}	mag_{I-band}
00034281002	2016-01-28	8	19.03±0.05	18.71±0.05	18.52±0.04	19.57±0.07
00034281003	2016-02-01	12	19.14±0.06	18.75±0.07	18.74±0.08	-
00034281004	2016-02-02	13	19.26±0.05	18.94±0.05	18.65±0.04	-
00034281005	2016-02-03	14	19.34±0.08	19.00±0.08	18.69±0.06	-
00034281006	2016-02-08	19	19.27±0.08	19.27±0.08	18.81±0.09	19.45±0.06
00034281007	2016-02-22	33	19.43±0.07	19.11±0.07	18.84±0.06	19.36±0.06
00034281008	2016-02-24	35	19.44±0.07	18.99±0.07	18.93±0.06	19.61±0.08
00034281009	2016-02-27	38	19.70±0.11	19.29±0.12	19.06±0.09	19.79±0.11
00034281010	2016-03-02	42	19.36±0.08	18.99±0.08	18.88±0.07	19.91±0.10
00034281011	2016-03-10	50	19.41±0.06	19.09±0.06	19.05±0.05	-
00034281012	2016-03-14	54	19.40±0.06	19.10±0.06	19.17±0.07	-
00034281013	2016-03-19	59	19.53±0.06	19.25±0.06	19.12±0.05	-
00034281014	2016-05-25	126	19.95±0.08	19.69±0.09	19.69±0.09	-
00034281015	2016-06-05	137	19.87±0.09	19.74±0.10	19.79±0.12	-
00034281016	2016-06-08	140	20.20±0.16	20.23±0.20	19.82±0.28	21.49±0.54
00034281018	2016-06-16	148	20.24±0.18	20.11±0.21	19.76±0.17	-
00034281019	2016-06-17	149	19.77±0.12	19.97±0.16	20.08±0.18	-
00034281020	2016-06-21	153	20.05±0.08	19.94±0.10	19.68±0.09	21.96±0.58
00034281021	2016-11-17	302	20.75±0.08	-	-	-
00034281024	2016-12-18	333	20.90±0.15	-	-	-
00034281025	2016-12-27	342	20.71±0.10	-	-	-
00034281026	2017-01-04	350	20.68±0.07	-	-	-
00034281027	2017-02-19	397	-	-	20.58±0.13	-
00034281028	2017-02-22	400	-	20.46±0.15	-	-
00034281029	2017-02-23	401	-	20.64±0.13	-	-
00034281030	2017-05-31	497	20.93±0.26	21.15±0.32	$>$20.88	-
00034281031	2017-06-04	501	20.72±0.14	20.64±0.16	20.40±0.15	-
00034281032	2020-02-09	1481	20.92±0.18	20.46±0.21	20.25±0.18	-

Notes. The days refer to the time relative to optical peak. Magnitudes (mag) are referred to the AB system.

Supplementary Table 2: Host Photometric Data of OGLE16aaa.

Telescope	Band	Flux Density	Unit
GALEX	1515Å (FUV)	6.8 ± 2.0	μJy
GALEX	2273Å (NUV)	16.9 ± 1.9	μJy
APM	4680Å (b_J)	82.5 ± 9.6	μJy
UVOT	1928Å (UVW2)	15.5 ± 2.6	μJy
UVOT	2247Å (UVM2)	23.8 ± 4.6	μJy
UVOT	2600Å (UVW1)	28.8 ± 4.8	μJy
UVOT	3465Å (U)	43.3 ± 7.6	μJy
UVOT	4392Å (B)	112.7 ± 14.5	μJy
UVOT	5486Å (V)	151.4 ± 37.6	μJy
2MASS	1.23μm (J)	0.42 ± 0.06	mJy
2MASS	1.67μm (H)	0.62 ± 0.08	mJy
2MASS	2.16μm (K_s)	0.55 ± 0.09	mJy
WISE	3.4μm (W1)	0.38 ± 0.01	mJy
WISE	4.6μm (W2)	0.31 ± 0.01	mJy
WISE	11.6μm (W3)	2.39 ± 0.13	mJy
WISE	22.1μm (W4)	3.37 ± 1.18	mJy

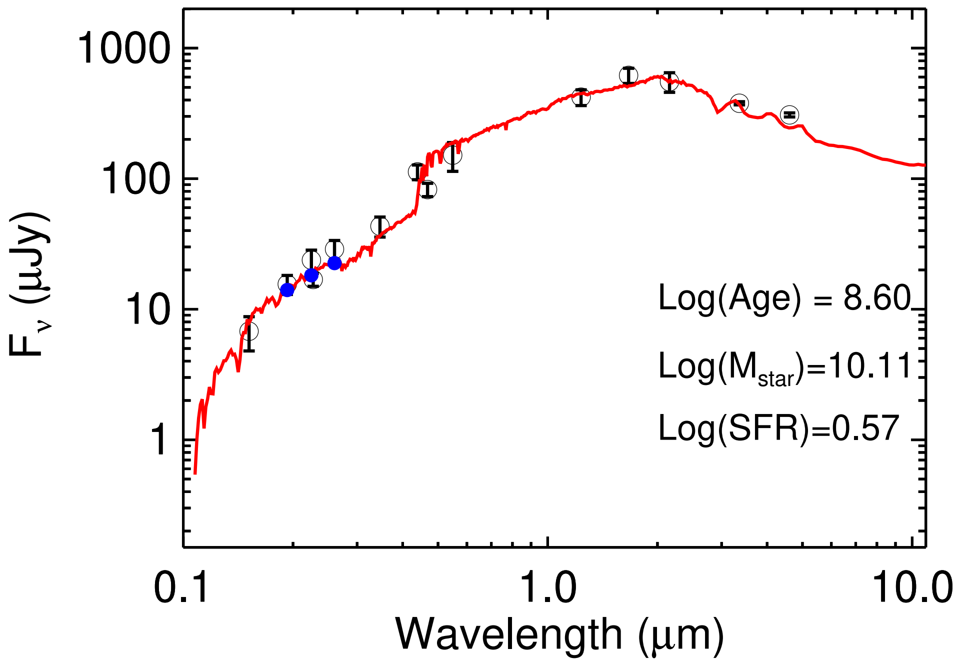

Supplementary Figure 1: SED fit for the host galaxy of OGLE16aaa. To perform the fit we use the code `FAST` and photometric data from the far-UV (GALEX) to the mid-IR (WISE). Error bars represent 1σ uncertainties due to photometric errors. The best-fitting template is shown in red line, with the best-fit stellar age, mass and star-formation rate noted in the legend. The extrapolated flux densities for the three Swift UV bands from the best-fit SED are shown in solid blue circles.

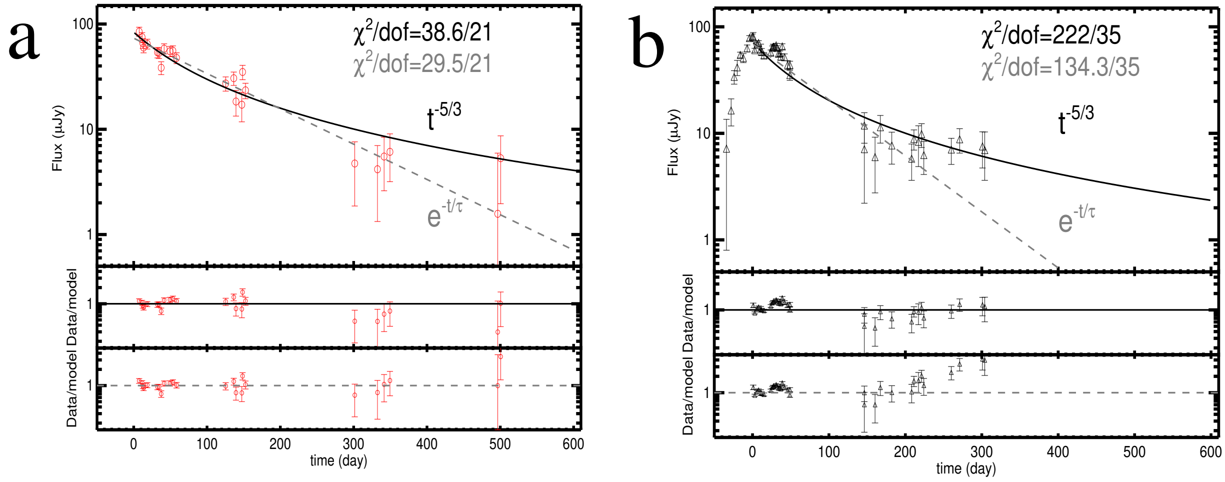

Supplementary Figure 2: Evolution of UV-optical emission at different epochs. **a** Evolution of UVW2 flux as a function of time, fitted with a $t^{-5/3}$ (solid line) and exponential decay model (dashed line), respectively. The corresponding data to model ratio is shown in the lower panel. **b** The same as **a**, but for the optical I-band data. Error bars correspond to the photometric uncertainties at the 1σ level.

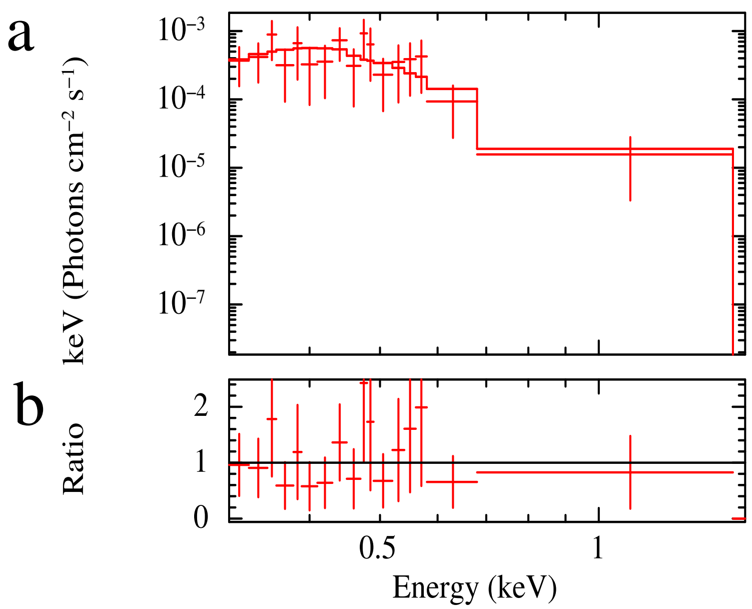

Supplementary Figure 3: Spectral fittings to the *Swift* data. **a** The combined *Swift* X-ray spectrum of OGLE16aaa observed at the peak (obsID 00034281019 and 00034281020) for a total exposure of 2750 sec. Error bars represent 1σ uncertainties calculated using Poisson statistics. Totally 40 ± 6 net counts are detected in the 0.3–2 keV. The spectrum can be well described by an absorbed blackbody model with $N_{\rm H} < 7.4 \times 10^{21}$ cm^{-2} and $kT = 73^{+22}_{-32}$ eV, which are consistent with the results of XMM-Newton observations within errors. **b** Data to model ratios.

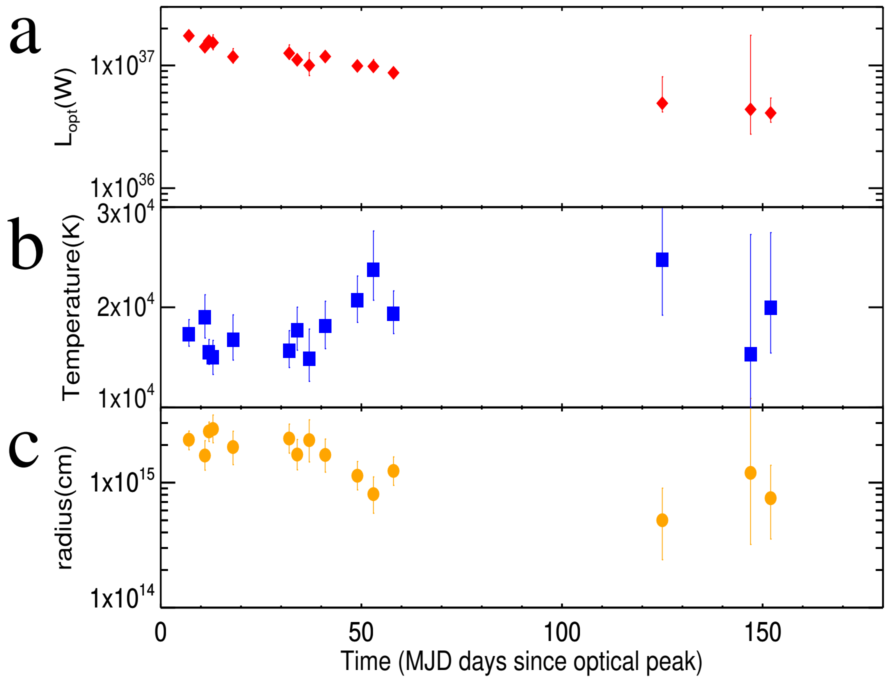

Supplementary Figure 4: The evolution of UV emission of OGLE16aaa at different epochs. The evolution of blackbody luminosity (**a**), temperature (**b**) and radius (**c**), are derived from blackbody fittings to the Swift UV data (Supplementary Table 1). Note that we do not show the fitting results from three epochs in which the model parameters are not well constrained. The error bars on each parameter are derived from the 16th and 84th percentiles of the distribution of the corresponding values obtained in 1000 simulations.

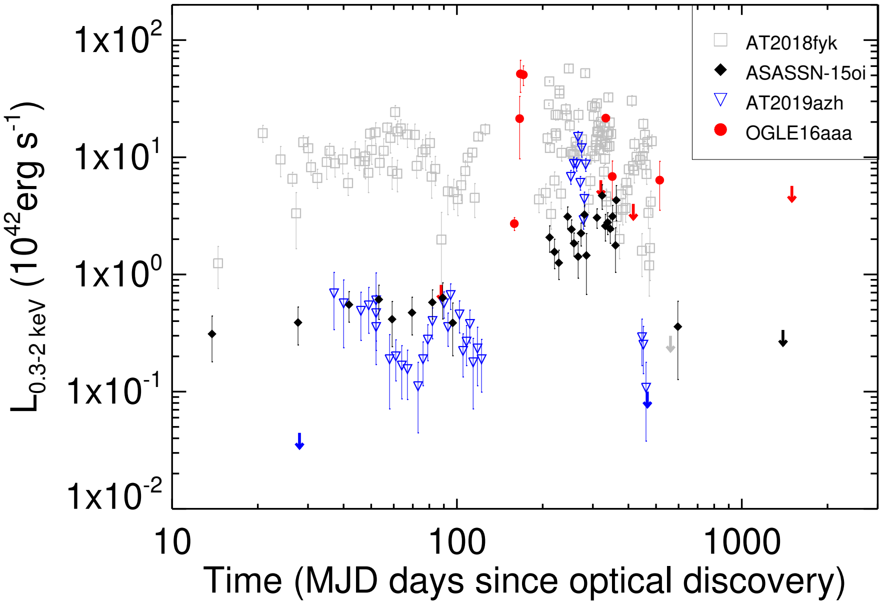

Supplementary Figure 5: The X-ray luminosity evolution of OGLE16aaa (red symbols). The luminosity evolution of optical TDE AT2018fyk, ASASSN-15oi and AT2019azh, are also plotted for a comparison, as noted in the legend. Error bars represent 1σ uncertainties calculated using Poisson statistics.

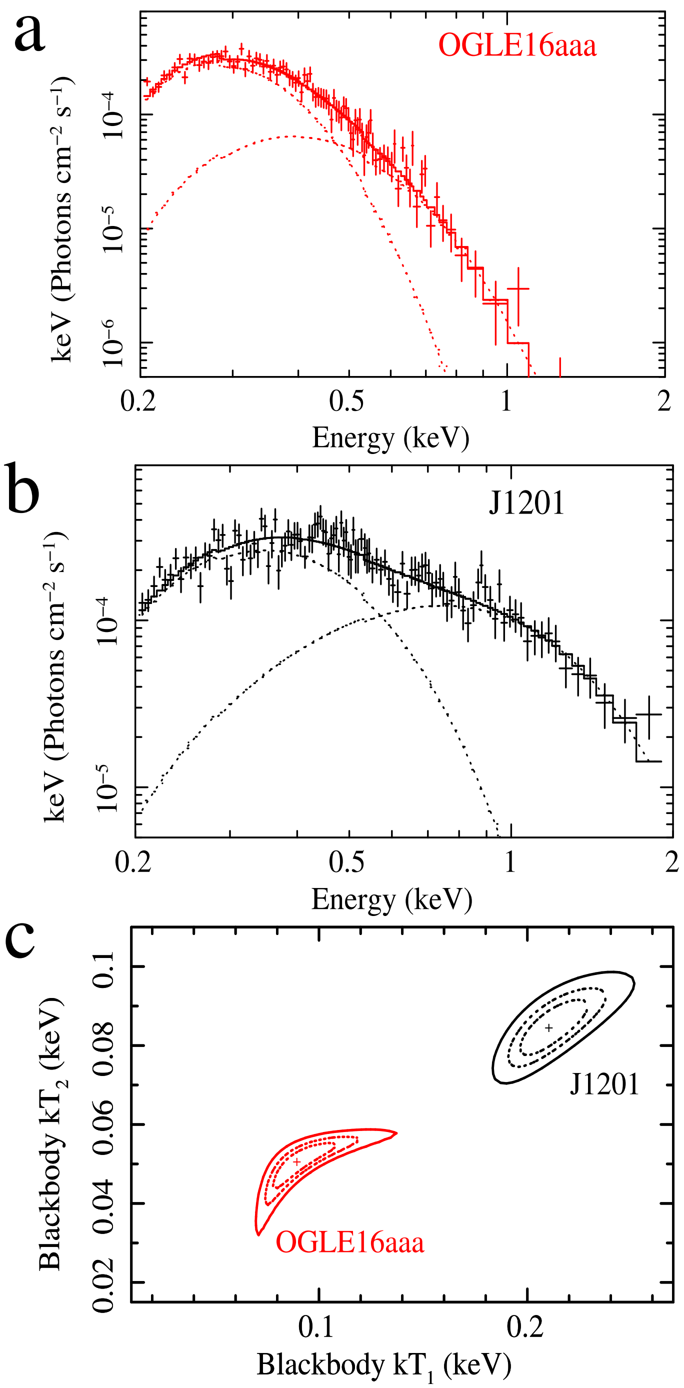

Supplementary Figure 6: Comparison of X-ray spectrum of OGLE16aaa with SDSS J120136.02+300305.5 (J1201). X-ray spectrum of OGLE16aaa and SDSS J120136.02+300305.5 is plotted in **a** and **b**, respectively, along with the spectral fitting results with double blackbody models. Error bars correspond to 1σ uncertainties calculated using Poisson statistics. **c** shows the joint 68%, 90% and 99% confidence contours of the corresponding blackbody temperatures. While the double blackbody models describe the data well, the resulted temperatures for the two TDEs are different.

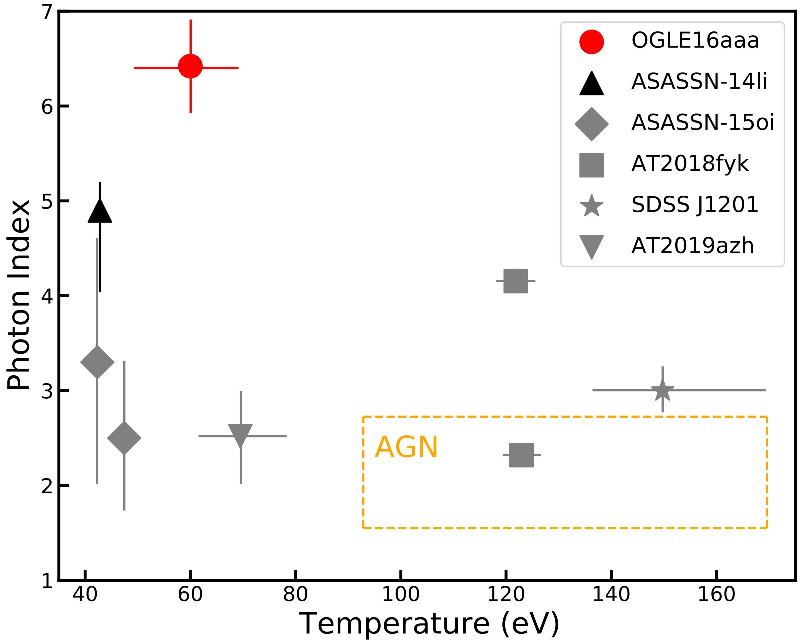

Supplementary Figure 7: Blackbody temperature versus photon index from the best-fitting model to the X-ray spectra of optical TDEs. Error bars correspond to 90% confidence intervals for one interesting parameter ($\Delta\chi^2 = 2.706$). The X-ray spectra are observed from XMM-Newton which have best S/N. The photon index represents the powerlaw model that is used to describe the additional X-ray component with respect to the primary blackbody emission. The region enclosed by the orange rectangle represents that for typical AGNs[6].

References

1. Kriek, M., van Dokkum, P. G., Labbé, I., et al. An Ultra-Deep Near-Infrared Spectrum of a Compact Quiescent Galaxy at z = 2.2. *Astrophys. J.*, **700**, 221 (2009).

2. Reines, A. E., & Volonteri, M. Relations between Central Black Hole Mass and Total Galaxy Stellar Mass in the Local Universe. *Astrophys. J.*, **813**, 82 (2015).

3. Wyrzykowski, Ł., Zieliński, M., Kostrzewa-Rutkowska, Z., et al. OGLE16aaa - a signature of a hungry supermassive black hole. *Mon. Not. R. Astron. Soc.*, **465**, L114 (2017).

4. Saxton, R. D., Read, A. M., Esquej, P., et al. A tidal disruption-like X-ray flare from the quiescent galaxy SDSS J120136.02+300305.5. *Astron. Astrophys.*, **541**, A106 (2012).

5. Liu, F. K., Li, S., & Komossa, S. A Milliparsec Supermassive Black Hole Binary Candidate in the Galaxy SDSS J120136.02+300305.5. *Astrophys. J.*, **786**, 103 (2014).

6. Crummy, J., Fabian, A. C., Gallo, L., et al. An explanation for the soft X-ray excess in active galactic nuclei. *Mon. Not. R. Astron. Soc.*, **365**, 1067 (2006).

7. Kara, E., Dai, L., Reynolds, C. S., et al. Ultrafast outflow in tidal disruption event ASASSN-14li. *Mon. Not. R. Astron. Soc.*, **474**, 3593 (2018).

8. Liu, X.-L., Dou, L.-M., Shen, R.-F., et al. The UV/optical peak and X-ray brightening in TDE candidate AT2019azh: A case of stream-stream collision and delayed accretion. Preprint at https://arxiv.org/abs/astro-ph/1912.06081 (2019).

9. Sun, L., Shu, X., & Wang, T. RX J1301.9+2747: A Highly Variable Seyfert Galaxy with Extremely Soft X-Ray Emission. *Astrophys. J.*, **768**, 167 (2013).
